# Integrating 5S Methodology into Oral Hygiene Practice for Elderly with Alzheimer’s Disease

**DOI:** 10.3390/dj8020029

**Published:** 2020-03-26

**Authors:** Sherry Shiqian Gao, Chun Hung Chu, Fanny Yuk Fun Young

**Affiliations:** 1 Faculty of Dentistry, The University of Hong Kong, Hong Kong 999077, China; sherryg@hku.hk (S.S.G.); chchu@hku.hk (C.H.C.); 2 Department of Business Administration, Hong Kong Shue Yan University, Hong Kong 999077, China

**Keywords:** 5S, dementia, Alzheimer’s disease, elderly, oral hygiene, dental caries, community dental care

## Abstract

Dementia is a clinical syndrome of loss of intellectual capability. Alzheimer’s disease (AD) is the dominant subtype of dementia and is common among the elderly. Because of impaired memory and disturbed executive functioning, the elderly with AD often have difficulty to perform oral hygiene practice and are at high risk of dental caries and periodontal diseases. These dental problems are ambulatory care-sensitive conditions where effective community dental care can help prevent the need for hospital admission. Community dental care practitioners can formulate effective strategies for the elderly with AD to reduce their risk of dental diseases. One of these strategies is to integrate 5S into oral hygiene practice. 5S was originally developed for organising spaces for people to work efficiently, effectively, and safely. It consists of five steps which are (i) sorting to remove unnecessary items, (ii) setting-in-order to place the items in order of flow, (iii) shining to clean and maintain the environment, (iv) standardising to establish discipline for good oral hygiene habits, and (v) sustaining to keep 5S going by auditing and improving the environment and oral hygiene practice. This system helps the elderly with AD to put things where they belong and keep the workplace clean. Moreover, it facilitates the elderly with AD to perform oral hygiene practice without wasting time and risking injury. This paper discusses the use of 5S to help the elderly with AD build and sustain an effective oral hygiene practice habit to improve their oral health.

## 1. Introduction

Dementia is a group of clinical syndromes characterised by the loss of intellectual capability to the extent that social or occupational functioning is impaired [1]. It is a broad category of brain diseases that cause an irreversible long-term and often gradual decrease in the ability to think and remember—a situation that is severe enough to affect daily functioning. People with dementia are conscious and have normal sensory functions. However, they can experience multiple cognitive deficits, such as the impairment of memory, the failure to recognise objects or persons, the deterioration of their language functions, and a disturbance in their executive functioning [2]. Moreover, people with dementia often have psychiatric and behavioural changes, such as personality changes, depression, and agitation.

The diagnosis of dementia is generally made based on the medical history of the disease and cognitive testing, with medical imaging and blood tests used to rule out other possible causes. Dementia is categorised as a neurocognitive disorder in the *Diagnostic and Statistical Manual of Mental Disorders* published by the American Psychiatric Association [2]. Neurocognitive disorders can be classified as major or minor. This sub-classification is used to reduce the stigma associated with dementia and to bring the diagnostic guidelines into alignment with current clinical practice [1]. Unfortunately, no known cure for dementia currently exists.

Dementia is common among the elderly, with an estimated 5% to 7% of elderly aged 60 years or above suffering from dementia in most regions of the world [3]. The GBD 2015 Disease and Injury Incidence and Prevalence Collaborators [4] reported that dementia affected approximately 46 million people worldwide in 2015. Because the incidence of dementia has been increasing exponentially with increasing age and with an aging population, the prevalence of dementia will probably be almost double, affecting 66 million people, in 2030 [3]. Dementia can be classified into various subtypes according to the underlying brain pathologies. The most common four subtypes include Alzheimer’s disease (AD), vascular dementia, dementia with Lewy bodies, and frontotemporal dementia [2].

## 2. Alzheimer’s Disease

AD is the most common subtype of dementia among the elderly, accounting for 50% to 75% of all dementia cases [5]. AD is a chronic neurodegenerative disease that irreversibly and progressively damages the brain. Common symptoms include problems with language, disorientation, not managing self-care, loss of motivation, and mood swings. Elderly with AD will gradually lose their memory and thinking skills, and, eventually, they will lose the ability to carry out the simplest tasks. Studies found that AD is associated with the deposition of amyloid beta, neurofibrillary tangles, neuronal loss in the brain, and genetic factors. However, the etiology of AD is unknown [5]. Clinical diagnosis is based on the medical history and on cognitive testing with medical imaging and blood tests to rule out other possible causes. A definite diagnosis often requires the examination of brain tissue. At present, no treatment is available to halt or reverse its progression.

## 3. Oral Health of the Elderly with Alzheimer’s Disease

Elderly with AD often suffer from upper respiratory tract infections and pneumonia, which is often related to their poor oral health. Although oral hygiene is important, their oral hygiene practice deteriorates with the progression of AD. Studies reported that elderly with AD have a high prevalence and risk of dental caries [6] and periodontal disease [7]. Their submandibular salivary output is impaired, and their resting salivary secretion is reduced [2]. Many of them have salivary dysfunction, which can be one of the side effects of their medication, such as cholinesterase inhibitors. The salivary dysfunction also increases their risk of burning mouth syndrome and opportunistic infection, such as oral candidiasis. A study found that elderly with AD who wore dentures had an increased prevalence of denture-related mucosal lesions, increased plaque accumulation, and an increased number of decayed retained roots [6].

Dental caries and periodontal disease are common oral health problems in elderly with AD. Oral health is an integral part of general health. Poor oral health affects the chewing ability and causes poor dietary intake and weight loss. In addition, poor oral health can adversely affect a person’s quality of life, overall health, and wellbeing. Good oral health starts with clean teeth. Tooth brushing is important for removing dental plaque, which is the primary cause of dental caries and periodontal disease. It also arrests the buildup of plaque formation. Unfortunately, because people with dementia always present symptoms like impaired memory and disturbance of behaviours, it is always challenging for them to perform appropriate and effective oral hygiene practices. Therefore, elderly with dementia often have poor oral hygiene, and therefore more bacteria in the oral cavity [2]. The oral health problems that elderly with AD face are ambulatory care-sensitive conditions, but effective community care and case management can help to prevent the need for hospital admission. Avoiding unnecessary hospitalisations is an important indicator of the quality of ambulatory care and the efficiency of the health system. Therefore, improving the oral health of elderly with AD can reduce the high prevalence of hospitalisations for ambulatory care-sensitive conditions.

## 4. Community Dental Care for Elderly with Alzheimer’s Disease

Although many elderlies with AD do not regularly attend dental clinics to monitor their oral health, they interact with various health care workers who can monitor and evaluate their health and assist in their personal care. These health care workers provide community care services aimed at helping people who need care and support to live with dignity and independence in the community and to avoid social isolation. They provide needy people with help at home and with support in getting out into the community. Furthermore, they can identify risks and help to eliminate or minimise risks where possible. Under the umbrella of community care, community dental care practitioners provide effective primary dental care to maintain or improve oral health for elderly with AD. Two of the main objectives of community dental care are to allow the timely use of personal oral health services to achieve the best possible health outcomes, and to prevent ambulatory care-sensitive conditions arising from dental problems. Community dental care practitioners can provide routine oral health assessments, oral health care planning, and support with daily oral care to elderly with AD. In addition, they can refer elderly with AD to dentists for dental care. Community dental care practitioners play an important role in developing the oral health knowledge and skills needed to support frail elderly. Hence, they formulate oral health care strategies and provide primary dental care for elderly with AD to reduce their risk of dental diseases. However, elderly with AD often have difficulty in performing oral hygiene practice due to their impaired memory and disturbed executive function. To deal with this challenge, community dental care practitioners can promote the oral health of elderly with AD using 5S for oral hygiene practice.

## 5. Improving Oral Hygiene Practice Using 5S

The Japanese originally developed 5S to establish and maintain a quality environment in an organisation [8]. 5S comes from five Japanese words: seiri (sort), seiton (set in order), seiso (shine), seiketsu (standardise), and shitsuke (sustain). 5S is useful for organising spaces where people can work efficiently, effectively, and safely. It consists of five steps: (i) sorting to remove unnecessary items, (ii) setting in order to place the items in order of flow, (iii) shining to clean and maintain the environment, (iv) standardising to establish discipline for good oral hygiene habits, and (v) sustaining to keep 5S going by auditing and improving the environment and oral hygiene practice. 5S helps elderly with AD to put the necessary items where they belong to and keep the workplace clean. Moreover, it helps elderly with AD to practice oral hygiene without wasting time and risking injury.

Step 1—Sorting

**Figure 1:**
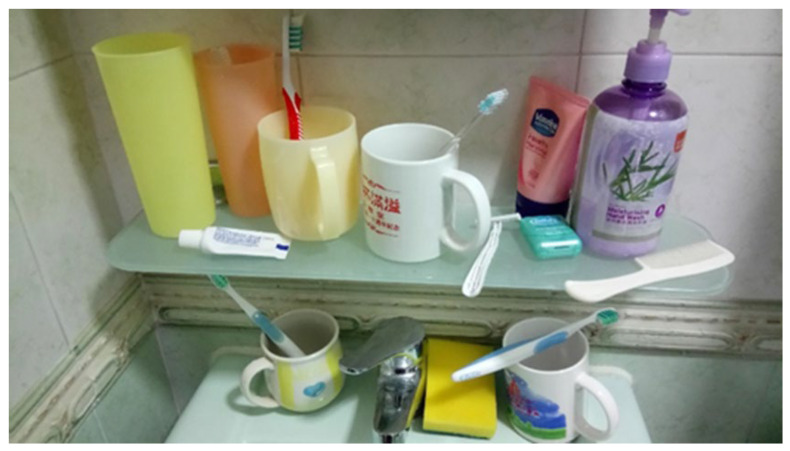
An untidy oral hygiene practice station in the bathroom.

The first step in the process is sorting. Sorting is about categorising items in the work environment. The oral hygiene practice stations (work environment) of elderly with AD are usually untidy (Figure 1). To help elderly with AD to perform oral hygiene practices, the first step is to sort out what is needed. The work areas, cabinets, and drawers should be cleaned, organised, and labelled according to their contents. This allows elderly with AD to identify the necessary items to do the work and to get rid of what is not needed. A photograph before sorting should be taken as a record (Figure 1). In the sample photograph, irrelevant items (hand wash, facewash, shaver, and comb) together with necessary items (toothbrushes, toothpaste, and rinsing cups) occupy the station for performing oral hygiene practices. During sorting, the necessary items or bathroom equipment for oral hygiene (e.g., toothbrush and rinsing cup) should be selected, and unneeded or useless equipment will be classified as unnecessary items and should be removed from the oral hygiene practice station.

Step 2—Setting in order

**Figure 2:**
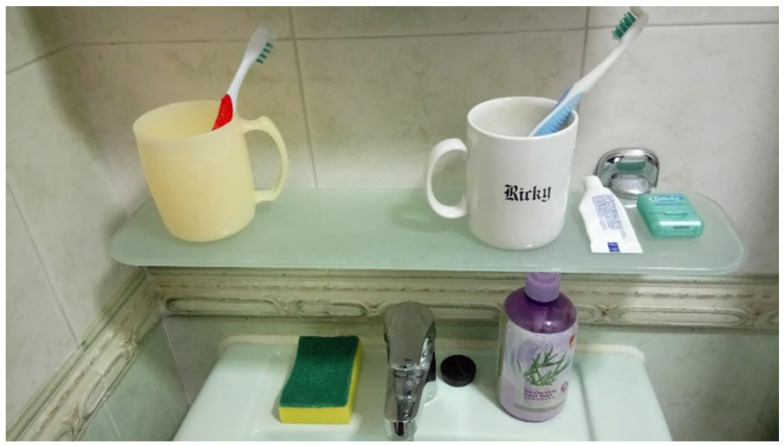
A tidy oral hygiene practice station with label on the elderly’s rinsing cup.

Setting in order is about creating a place for everything and putting everything in its place. Setting in order essentially involves putting away the things that elderly should not keep. After the first step, sorting items involves classifying them as needed or not needed. The next step is to get rid of what is not needed. The bathroom should be organised to make it an optimal place for oral hygiene practice. The oral hygiene equipment should be put in an accessible and convenient location, and other unnecessary items should be stored away. The setting in order principle is to arrange work items in a sequence of oral hygiene practice that relates to the work involved. Hence, the location of each piece of equipment should be fixed. The work area, storage area, and equipment are clearly marked and well organised. This makes oral hygiene practice as simple and straightforward as possible for people with AD. The reasons behind this are that items should be easier and faster to find, which can help to save both time and frustration. A photograph after sorting and setting in order (Figure 2) should be taken for use in the next step: shining.

Step 3—Shining

**Figure 3:**
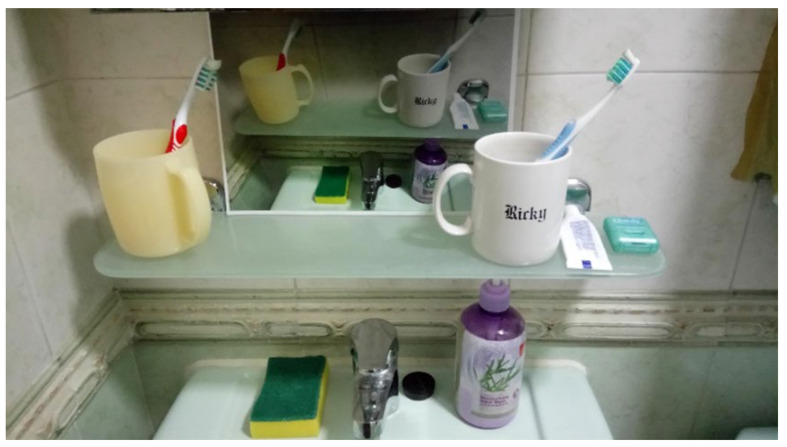
Using a photograph as a reminder of setting in order and shining.

The third step is shining. Shining is about cleaning and maintaining the environment. The principle of shining is to inspect and clean the work environment to improve the appearance of the workplace. After everything is sorted and put in order, we must clean. This is shining. When we clean, we can observe things that need to be fixed in the workstation. Shining can involve removing old notices and sticky tapes to clean the area, as well as removing broken equipment to avoid posing a safety risk. Shining also includes checking the work environment for good air quality, adequate lighting, and the absence of dust or fumes. The photograph of a tidy oral hygiene practice station can be stuck on the bathroom wall as a reminder (Figure 3). Elderly with AD should be trained to always put oral hygiene equipment in the precise location. It is important to instil the concept of cleanliness in elderly with AD. They should be educated and learn that the personal environment should be as clean as the bathroom environment, and the oral cavity should also be cleaned thoroughly as part of an oral hygiene routine.

Step 4—Standardising

The fourth step is standardising. This is about establishing discipline for good oral hygiene habits. After sorting, setting in order, and shining, we should stand back and look at the accomplishment. We should make an effort to build oral hygiene practices after creating a tidy environment for elderly with AD. In addition, we should instil the concept of cleanliness in elderly with AD. The oral hygiene practices should become a standard daily procedure carried out at a convenient and standard time. This involves training and continuously performing routine oral hygiene practices. Reminders of oral hygiene practices should be placed on the bathroom door to remind elderly with AD to brush after getting up. They should also be placed on the wall in front of the bed so that they can remember to brush before going to sleep.

Step 5—Sustaining

The fifth and final step is sustaining. Sustaining is about auditing and improving oral hygiene practices. The principle of sustaining involves recognising the results achieved through the previous four steps and maintaining a standardised new work environment. This can be the most difficult step, as it involves having discipline and keeping 5S processes going. The optimal environment for oral hygiene practices should be maintained. The previous four steps of building oral hygiene habits should be monitored, reviewed, and reinforced regularly every week. Moreover, the oral hygiene practices should be evaluated and revised for continuous improvement. Table 1 shows a 5S checklist that can be used to monitor the progress of the oral hygiene practices. This often involves the whole family working together to maintain and sustain these habits.

The above five steps of 5S describe how to create a new, effective, and efficient work environment, followed by how to maintain newly created oral hygiene practice behaviours for elderly with AD. This 5S oral hygiene practice program requires the effective communication and collaboration of the community dental care practitioner with elderly with AD and, if any, their caregivers and/or family members. The caregivers and/or family members can be empowered to guide and to monitor elderly with AD in performing 5S oral hygiene practices. They can also assist the community dental care practitioner in checking the progress and compliance so that a sustainable improvement in the oral hygiene practices of people with AD can be achieved. By adopting the 5S oral hygiene practices, people with AD are expected to develop regular and effective oral hygiene practices to maintain their oral health, which is an essential part of their general health.

## 6. Implementing 5S in Healthcare

5S is a problem-solving approach to improve efficiency in the work environment by removing unnecessary items, cleaning and clearing workplaces, and maintaining an effective work environment. It improves efficiency, safety, and morale due to improvements in the work environment. 5S can be a workplace organisation methodology for companies and organisations for continuous improvement, including healthcare organisations. The 5S strategy developed for healthcare organisations usually includes the following steps. The first step involves removing broken, out-of-date, or recalled items from the workplace to prevent the use of potentially harmful items (sorting). The second step involves clearing the path of storage, designing the storage ergonomically, then organising and storing items to make sure that the items can be accessed quickly and safely (setting in order). The third step involves ensuring that the items and equipment are sanitary or sterile so that they are ready for use (shining). The fourth step entails organising the rooms and clinics with similar functions to reduce the time and effort required to adapt to a new environment (standardising). The fifth and final step involves maintaining all the previous components, checking the quality, and ensuring continued compliance (sustaining). A study in a hospital reported that staff implementing the 5S strategy could (1) take fewer steps in admitting, treating, and discharging a patient; (2) spend more value-added time on face-to-face contact with the patient; and (3) produce less travel distance compared with before the adoption of the 5S strategy [9]. Another study found that the 5S strategy enhanced safety in clinical practices in different ways, such as the cleanup of expired or obsolete items, contamination prevention, and the standardisation of cleaning and sterilising procedures [10].

Healthcare costs have been increasing over the years. The increased cost will not only bring pressure to healthcare providers but also it will place a burden on the social welfare system and on the government. Reducing costs and maintaining service quality and safety at the same time are always challenging tasks for healthcare providers [11]. A number of hospitals and government healthcare services have been using 5S to reduce costs, improve quality, and subsequently enhance cost effectiveness and efficiency [9,10,11]. 5S provides a number of benefits to the healthcare system, including the improvement of the utilisation of resources and spaces, the reduction of the leading time and cycle time within processes, saving costs related to both materials and the workforce, and the enhancement of services’ quality and safety. This ultimately helps to reduce the burden placed on the social welfare system and on the government.

## 7. Conclusions

Dental caries and periodontal disease are common in elderly with AD. These dental problems are ambulatory care-sensitive conditions, where effective community dental care can help to prevent the need for hospital admission. 5S is a problem-solving approach to helping elderly with AD to build and sustain effective oral hygiene practices for improving their oral health.

## Figures and Tables

**Table 1:** 5S checklist of sustained oral hygiene practices.

No.	Activities	Check/Finding	Action by (Date)
1	Remove unnecessary things in the oral hygiene practice station.		
2	Organise necessary equipment for oral hygiene practices in the station.		
3	Clean up toothbrushes and cups. Replace used-up toothpaste and dental floss.		
4	Manage defects, leakage, and breakage found in the oral hygiene practice station.		
5	Take a photo of a tidy oral hygiene practice station.		
6	Compare the photo with the actual condition. Check the condition and location of the equipment.		
7	Check the reminder posted on the walls. Check if the elderly is aware of the reminder.		
